# Commercial Payments for COVID-19–Associated Inpatient Stays in 2020

**DOI:** 10.1001/jamahealthforum.2023.3711

**Published:** 2023-11-10

**Authors:** Rose Kerber, Erin L. Duffy, Christopher Whaley

**Affiliations:** 1 RAND Corporation, Boston, Massachusetts; 2 Leonard D. Schaeffer Center for Health Policy and Economics, University of Southern California, Los Angeles; 3 RAND Corporation, Santa Monica, California; 4 Department of Health Services, Policy, and Practice, School of Public Health, Brown University, Providence, Rhode Island

## Abstract

This cross-sectional study reports the allowed reimbursement amounts for inpatient COVID-19 care for different types of hospitals.

## Introduction

At the COVID-19 pandemic onset in the US in March 2020, the overall volume of hospital inpatient admissions decreased despite the influx of COVID-19–associated admissions.^1^ Decreases in non–COVID-19 care contributed to reductions in hospital finances and subsequent federal financial assistance to hospitals.^2,3,4^ While Medicare and public insurers have posted reimbursement rates for COVID-19 care, payment rates and sources of rate variation among patients with commercial insurance are mostly unknown, despite many patients with COVID-19 having commercial coverage, thus affecting hospital finances. We examined commercial payment for inpatient COVID-19 care in 2020 in a convenience sample of commercial insurers across the US.

## Methods

This cross-sectional study used 2020 commercial medical claims from 11 state all-payer claims databases and over 100 self-insured employers with representation from all 50 US states (eAppendix in Supplement 1).^5^ COVID-19–associated inpatient stays were identified from inpatient claims with a primary *International Statistical Classification of Diseases, Tenth Revision, Clinical Modification* (*ICD-10-CM*) diagnosis code of U07.1. The RAND Institutional Review Board approved the study and waived the informed consent requirement because deidentified data were used. We followed the STROBE reporting guideline.

Allowed amounts incorporate both facility and professional services provided during each inpatient stay and capture the transacted amount of each service, including payments by patients and insurers. For each inpatient stay, the Medicare reimbursement rate for the services was tabulated, and the ratio of commercial to Medicare allowed amounts was calculated. Multivariate generalized linear model regression was used to assess the association between commercial allowed amounts for COVID-19–associated stays and hospitals’ for-profit, teaching, or health system affiliation; Centers for Medicare & Medicaid Services Star Rating (4-5 stars); and safety-net (disproportionate share hospital) status, adjusting for patient age, sex, length of stay (LOS), and region. Results were converted to adjusted absolute dollar differences.

Two-sided *P* = .05 indicated statistical significance. Analyses were conducted using Stata 16 (StataCorp LLC).

## Results

The sample included 12 183 inpatient stays at 1760 hospitals for commercially insured patients with an *ICD-10-CM* U071 diagnosis code. Patients had a mean (SD) age of 51.2 (10.5) years; comprised 6582 males (54.0%) and 5595 females (46.0%); and had a mean (SD) LOS of 6.0 (6.5) days (Table 1).

**Table 1.** Patient and Hospital Characteristics^a^

Characteristic	No. (%)
**Patients**	
LOS, mean (SD), d	6.0 (6.5)
Age, mean (SD), y	51.2 (10.5)
Sex	
Male	6582 (54.0)
Female	5595 (46.0)
**Hospitals** ^b^	
Teaching hospital	7979 (65.5)
For-profit hospital	1336 (11.0)
High-quality hospital: 4 or 5 CMS Star Rating	5868 (48.2)
Safety net hospital or DSH	4689 (38.5)
Health system–affiliated hospital	7446 (61.1)
Census region	
Midwest	1854 (15.2)
Northeast	2360 (19.4)
South	4002 (32.9)
West	3965 (32.6)
Allowed amount, mean (SD), $	$32 985 ($51 995)
Claims per stay	11.7 (16.8)
Services	12 183

Abbreviations: CMS, Centers for Medicare & Medicaid Services; DSH, disproportionate share hospital; LOS, length of stay.

^a^ Descriptive characteristics of the study sample of COVID-19-associated inpatient stays in 2020 covered by commercial insurance. Sample represented observations from all 50 US states.

^b^ Teaching status, hospital profit status, DSH information obtained from CMS 2021 Hospital Cost Report data; CMS Star Rating data from CMS HospitalCompare website; and hospital system affiliation from the Agency for Healthcare Research and Quality 2018 Compendium of US Health Systems.

The mean (SD) commercial allowed amount for COVID-19–associated inpatient stays was $32 985 ($51 995). The distribution of commercial allowed amounts was positively skewed, with a median (IQR) of $21 190 ($14 583-$32 182). The mean (SD) and median (IQR) ratios of commercial to Medicare allowed amounts were 2.43 (1.79) and 2.14 (1.46-2.99), respectively. The mean (SD) allowed amount per day was $7895 ($14 134), with a median (IQR) of $5216 ($3364-$8152).

After adjustment for patient age, sex, number of claims, and LOS, the commercial allowed amounts for COVID-19–associated inpatient stays were higher for hospitals affiliated with a health system owning 5 or more acute hospitals ($3562; 95% CI, $1702-$5422) and hospitals with a 4 or 5 star rating ($2931; 95% CI, $912-$4949) (Table 2). For-profit ownership also had a large, but not statistically significant, estimated difference.

**Table 2.** Association Between Commercial Allowed Amounts and Patient and Hospital Characteristics

Characteristic	Regression-adjusted allowed amounts (95% CI), $	*P* value
**Patients**		
LOS, d	1200 (1003 to 1398)	<.001
Age	62 (–13 to 137)	.11
Male sex	924 (–773 to 2621)	.29
Female sex	1 [Reference]	
No. of claims	254 (189 to 319)	<.001
**Hospitals**		
Teaching hospital	1909 (–60 to 3877)	.06
For-profit hospital	2848 (−626 to 6323)	.11
High-quality hospital: 4 or 5 CMS Star Rating	2931 (912 to 4949)	.004
Safety net hospital or DSH^a^	2071 (257 to 3885)	.03
Health system–affiliated hospital^b^	3562 (1702 to 5422)	<.001
Census region		
Midwest	1 [Reference]	
Northeast	5326 (2157 to 8315)	.001
South	–6333 (–9062 to –3604)	<.001
West	2484 (–211 to 5179)	.07

Abbreviations: CMS, Centers for Medicare & Medicaid Services; DSH, disproportionate share hospital; LOS, length of stay.

^a^ Safety-net hospital defined as a hospital with allowable DSH discharges at or above 15% based on hospital cost reporting; data from 2021 RAND hospital data sets.

^b^ Health system–affiliated hospital defined as a hospital belonging to a health system that owns 5 or more acute care hospitals; data from Agency for Healthcare Research and Quality 2018 Compendium of US Health Systems.

## Discussion

This cross-sectional study found that commercial payments for inpatient COVID-19 care in 2020 were higher among system-affiliated hospitals and high-quality hospitals, with the largest differences among for-profit hospitals. Ratios of commercial to Medicare allowed amounts were similar in magnitude between COVID-19–associated and other inpatient stays.^5^ Study limitations included use of select commercial claims data and exclusion of noncommercial claims, such as Medicaid or uninsured populations.

Consistent with studies of pricing for other health care services, this study found that system-affiliated, for-profit, and high-quality hospitals had higher reimbursable amounts, despite adjustments for patient LOS and age. As policymakers seek to provide equitable financial assistance to hospitals, it is important to recognize that hospitals received widely varied amounts for COVID-19 care and, when appropriate, to adjust for differences in payment rates to ensure that hospitals with less resources receive adequate assistance.

## Notes

Supplement 1.
eAppendix

eReference

Supplement 2.
Data Sharing Statement
