# Supplementary material for: Commercial Payments for COVID-19–Associated Inpatient Stays in 2020
Source: JAMA Health Forum. 2023 Nov 10;4(11):e233711. doi: 10.1001/jamahealthforum.2023.3711 (PMC10638639; doi:10.1001/jamahealthforum.2023.3711)
Supplement: Supplement 1. — eAppendix eReference [file jamahealthforum-e233711-s001.pdf]

Supplementary Online Content

Kerber R, Duffy EL, Whaley C. Commercial payments for COVID-19–associated inpatient stays in 2020. *JAMA Health Forum.* Published online November 10, 2023. doi:10.1001/jamahealthforum.2023.3711

eAppendix
eReference

This supplementary material has been provided by the authors to give readers additional information about their work.

© 2023 Kerber R et al. *JAMA Health Forum.*

eAppendix

This study used 2020 medical claims data from the RAND Hospital Price Transparency Project, Round 4.[1] This study collected data from several self-funded employers and state all-payer claims data bases. Self-funded employers were recruited using a snowball sampling approach. Participating employers included a variety of industries ranging from state and local governments, manufacturing, to higher education. Participating employers also in the number of covered lives, from a few hundred to more than 100,000. The employer data used in the RAND transparency study included employers from all U.S. states. It also included both employers who operate within a single market and employers with a national presence. Finally, the employer data also included data from several state employee plans. In many states, the state employee health plan is the largest provider of health insurance benefits.

The second source of data included all-payer claims data (APCD) from eleven states—Arkansas, Delaware, Colorado, Connecticut, Maine, New Hampshire, Oregon, Rhode Island, Utah, Vermont, and Washington. APCD data was limited to commercial insured populations, excluding Medicare Advantage and Medicaid managed care plans. APCD data includesd a mix of individual (primarily ACA Exchange plans), fully insured, and self-insured populations. Due to the U.S. Supreme Court's *Gobeille v. Liberty Mutual Insurance Company* decision, non-municipal participation in state APCDs is voluntary, and states vary in their ability to include data from *ERISA*-regulated self-funded plans.

For both sources of medical claims data, data included claim identifiers and line item–level detail on services provided and allowed amounts. We used the claim and line-item identifiers to group claims into DRG procedures. COVID-19- related inpatient stays were identified as claims with a primary diagnosis code of U071 on an inpatient claim. Consistent with Medicare payment policies, we applied a 20 percent increase to the DRG weight for COVID inpatient claims.

eReference

1. Whaley CM, Briscombe B, Kerber R, O'Neill B, Kofner A. *Prices Paid to Hospitals by Private Health Plans: Findings from Round 4 of an Employer-Led Transparency Initiative*. RAND Corporation; 2022. Accessed June 9, 2022. https://www.rand.org/pubs/research_reports/RRA1144-1.html

© 2023 Kerber R et al. *JAMA Health Forum.*
